# Antimicrobial and Antioxidant Activity of Different Honey Samples from Beekeepers and Commercial Producers

**DOI:** 10.3390/antibiotics11091163

**Published:** 2022-08-29

**Authors:** Miroslava Kačániová, Petra Borotová, Lucia Galovičová, Simona Kunová, Jana Štefániková, Przemysław Łukasz Kowalczewski, Peter Šedík

**Affiliations:** 1 Institute of Horticulture, Faculty of Horticulture and Landscape Engineering, Slovak University of Agriculture, Tr. A. Hlinku 2, 94976 Nitra, Slovakia; 2 Department of Bioenergy, Food Technology and Microbiology, Institute of Food Technology and Nutrition, University of Rzeszow, Zelwerowicza St. 4, 35601 Rzeszow, Poland; 3 AgroBioTech Research Centre, Slovak University of Agriculture, Tr. A. Hlinku 2, 94976 Nitra, Slovakia; 4 Institute of Food Sciences, Faculty of Biotechnology and Food Sciences, Slovak University of Agriculture, Trieda A. Hlinku 2, 94976 Nitra, Slovakia; 5 Department of Food Technology of Plant Origin, Poznań University of Life Sciences, 31 Wojska Polskiego St., 60-624 Poznań, Poland; 6 Institute of Marketing, Trade and Social Sciences, Faculty of Economics and Management, Slovak University of Agriculture, Trieda A. Hlinku 2, 94976 Nitra, Slovakia

**Keywords:** bee honey, beekeepers, commercial, antimicrobial activity, antioxidant activity, bacteria, yeasts

## Abstract

Honey contains compounds with antioxidant and antibacterial capacities, such as phenolic compounds and carotenoids. The current analysis evaluates the antioxidant and antibacterial activity of 100 honey samples from beekeepers from Slovakia and commercially purchased ones. Honey samples were diluted to 50%, 25%, 12.5%, and 6.25% concentrations. The antimicrobial activity of honey samples was evaluated against three Gram-positive, three Gram-negative bacteria, and four *Candida* spp. by well diffusion method. The highest antimicrobial effect of all honey concentrations was expressed as the size of the inhibition zone and was found against *Pseudomonas aeruginosa* among Gram-negative bacteria, *Bacillus subtilis* among Gram-positive bacteria, and *Candida tropicalis* among yeasts. Antibiotics used in the study showed the highest antimicrobial effect compared to all concentrations of honey samples. Slovakian honey from beekeepers and commercial honey samples from the Slovak market showed variable inhibitory effectiveness against microorganisms. The honey concentration of 50% was found the most effective. Lower concentrations of honey exhibited no effect against yeasts. The best antioxidant activity was found in a sample of buckwheat honey yielding 70.83% of DPPH inhibition and 2373.85 μg/g TEAC. Overall, better antioxidant activity was evaluated in honeydew honey.

## 1. Introduction

Honey is one of the major dietary components for humans, due to its therapeutic [1], antioxidant [2,3], antimicrobial [4,5], antitumoral [6], anti-inflammatory [7], antiviral [8], and antiulcer [5] activities.

The composition of honey, also in terms of the content of phenolic compounds, depends primarily on the botanical origin [9,10]. External factors, such as environmental conditions, harvesting season, storage, and processing method, also play an important role. Phenolic compounds are the most important antioxidants in honey. Besides having other effects, they are responsible for the therapeutic properties of honey which motivate its use in traditional and modern medicine for the treatment of human illnesses. Examples of the therapeutic applications of honey include facilitation of the treatment of diseases associated with oxidative stress, such as diabetes mellitus, hypertension, atherosclerosis, cancer, and Alzheimer’s disease [11]. In addition, due to the rich content of phenolic compounds in honey, the addition of honey may positively affect the organoleptic properties (color, taste, or flavor) of food [12]. Moreover, phenolic compounds were proposed as a chemical marker to determine the botanical and geographical origins of honey in a recent study [13]. 

Numerous studies focused on the antimicrobial potential of honey have been published to date [14,15,16]. The discovery of penicillin was one of the pivotal moments of modern medicine. However, with the rise of antibiotic-resistance in microbes, effectiveness of conventional antibiotics became threatened. Novel antibiotic-resistant forms have evolved within several bacterial genera, including *Staphylococcus*, *Enterococcus*, and *Mycobacterium*. Overuse of antibiotics makes the situation much worse by driving the occurrence of multidrug-resistant microbes [17,18]. Furthermore, high expenses associated with medication development hinder the production of new antimicrobial drugs to combat the emerging threat of antibiotic-resistant bacterial forms. 

The aim of this study was to evaluate the antioxidant and antibacterial effect of honey from beekeepers and commercial bee honey samples of different floral origins against six strains of bacteria and four strains of yeast.

## 2. Results

### 2.1. Antimicrobial Activity of 50% Honey Samples

A 50% concentration of honey was tested against Gram-negative bacteria and the range of inhibition zones was determined in Table 1, Table 2 and Table 3. The antimicrobial activity against *S. enterica* ranged between 0.00 and 10.67 mm. The biggest inhibition zones were created by honey samples no. 31 and 88. No 31. was a multifloral honey from a beekeeper collected from forest and no. 88 was a commercial honey from linden. Streptomycin had a stronger antimicrobial effect (20.67 mm) than honey samples against *S. enterica* (Table 1). In 8 samples, antimicrobial activity of the 50% honey concentration against *S. enterica* was not observed at all (no. 19, 20, 23–28). 

The antimicrobial activity of honey samples against *Y. enterocolitica* ranged between 0.00 and 10.67 mm. The highest value of inhibition was found in three samples, no. 31, 32, and 62. No. 31 was a multifloral honey collected from a forest and obtained from a beekeeper. No. 32 was also honey from a forest obtained from a beekeeper, but it was produced from honeydew. Sample no. 62 was honey of a rapeseed type from a countryside apiary obtained by a beekeeper. Also in this case, streptomycin had a stronger antimicrobial effect against *Y. enterocolitica* (14.67 mm) (Table 2). *Y. enterocolitica* was resistant against 53 honey samples where no antimicrobial activity was observed (Samples 1, 2, 4–12, 21–24, 34–37, 39–43, 49–52, 54–55, 69–76, 85–99).

The inhibition zones of honey samples created against *P. aeruginosa* ranged between 0.00 and 17.67 mm. The best antimicrobial activity was found in linden honey obtained from a beekeeper in the countryside. Antibiotic streptomycin resulted in a 20.33 mm inhibition zone against *P. aeruginosa* (Table 3). Sixty-eight honey samples did not show inhibitory effects against this microorganism (Samples no. 1–4, 13–31, 33–40, 47–49, 51–60, 67–68, 74–88, 90–96). 

The antibacterial activity of the 50% honey was compared against the Gram-negative bacteria mentioned. Against *S. enterica*, the activity was found in 92% of honey samples, against *Y. enterocolitica* in 57%, and against *P. aeruginosa* in 32% which suggests that the most vulnerable G^-^ bacteria was *S. enterica* and the most resistant was *P. aeruginosa*.

The sizes of the inhibition zones resulting from honey samples in the 25% concentration observed for Gram-negative bacteria are shown in Table 1, Table 2 and Table 3. The antimicrobial activity against *S. enterica* was observed only in 8 cases and ranged between 1.67 and 7.00 mm. The best antimicrobial activity was found in honey sample no. 31 which was a multifloral honey from a beekeeper obtained from the forest (Table 1). Among these honey samples, six were purchased from beekeepers and two were purchased from commercial shops.

The antimicrobial activity of the 25% honey concentration against *Y. enterocolitica* was found in 12 cases and inhibition zones ranged from 5.67 to 8.67 mm. The best result was observed in two samples, no. 29 was a honeydew honey from a beekeeper from a forest, and no. 61 a multifloral honey from a beekeeper from a city (Table 2). All samples which were effective in the 25% concentration were obtained from beekeepers.

The antimicrobial activity of the 25% concentration of honey samples against *P. aeruginosa* was found in 12 samples with zones ranging between 6.67 and 10.67 mm. The best antimicrobial activity was found in the linden honey obtained from a beekeeper in the countryside (Table 3). All samples that showed antimicrobial activity in the 25% concentration were obtained from beekeepers.

In the case of the 12.5% honey samples against Gram-negative bacteria, an antibacterial effect was found only against *P. aeruginosa* (Table 3). It was observed in 12 samples, and ranged between 4.33 and 8.33 mm. The best antimicrobial activity was found in multifloral honey from a beekeeper from a town. No antibacterial activity for the 6.25% concentration of honey samples was detected against Gram-negative bacteria.

The antimicrobial activity of honey samples against Gram-positive bacteria is shown in Table 4, Table 5 and Table 6. The inhibition zones created against *S. aureus* ranged between 0.00 and 12.33 mm (Table 4). The best antimicrobial activity was found in honey sample no. 3. It was honey from a beekeeper of multifloral origin collected in a town. Against *S. aureus*, a stronger antimicrobial effect was observed for the antibiotic chloramphenicol (22.33 mm) compared to honey samples (Table 4). In 82 samples, antimicrobial activity of the 50% honey against *S. aureus* was not observed. 

The antimicrobial activity of honey samples at the 50% concentration against *E. faecalis* showed inhibition zones which ranged between 0.00 and 12.33 mm (Table 5). This highest value was found in sample no. 16, in honey from a beekeeper of multifloral origin collected from a town. The results were similar compared to *S. aureus*, but a larger antimicrobial effect was determined with the antibiotic chloramphenicol against *E. faecalis* (24.67 mm). No antimicrobial activity against this microorganism was found in 16 samples. 

The antimicrobial activity of honey samples against *B. subtilis* ranged between 0.00 and 12.67 mm (Table 6). The best antimicrobial activity was found in chestnut honey from a beekeeper collected from the forest. In the case of chloramphenicol, the diameter of the inhibition zone was 22.33 mm. In total, 23 honey samples did not show an effect against *B. subtilis*. 

The antibacterial activity of the 50% honey against *S. aureus* was found only in 18% of honey samples, against *E. faecalis* in 84%, and against *E. faecalis* in 77%. This result implies the best activity of honey against *E. faecalis*. The ranges of the inhibition zones of 25% of honey samples in the tested Gram-positive bacteria are shown in Table 4, Table 5 and Table 6. Against *S. aureus*, 13 samples of honey showed antimicrobial activity which ranged between 2.38 and 13.00 mm. The best antimicrobial activity was found in honey sample no. 4, which was multifloral honey from a beekeeper from a town (Table 4). All antibacterial activities of the 25% honey against *S. aureus* were found in samples obtained from beekeepers.

The antimicrobial activity of honey samples against *E. faecalis* was found only in 4 samples of the 25% honey and ranged between 10.00 and 11.00 mm. The highest value was found only in sample no. 16, in multifloral honey from a beekeeper from a town (Table 5). All samples which were effective in the 25% concentration against *E. faecalis* were from beekeepers.

The antimicrobial activity of honey samples against *B. subtilis* was found in 9 samples and ranged between 4.33 and 14.33 mm. The best antimicrobial activity was found in sample no. 4, a multifloral honey from a town obtained from a beekeeper (Table 6). All samples which showed antimicrobial activity in the 25% concentration against *B. subtilis* were from beekeepers.

The antibacterial activity of the 12.5% honey was found against all three Gram-positive bacteria species. The antibacterial effect against *S. aureus* was observed in 9 samples with the best activity observed in sample no 4. It was a beekeeper’s honey of multifloral origin from a town (Table 4). Honey in the 6.25% concentration showed an antibacterial effect only in two samples. No. 4 had an inhibition zone of 5.33 mm (multifloral honey from a beekeeper from a town). Sample no. 53 had an inhibition zone of 3.33 mm (fruit-tree honey from a beekeeper from a countryside).

The antimicrobial effects of the 12.5% honey against *E. faecalis* were found in four samples and ranged between 5.67 and 8.33 mm. The best antimicrobial activity was observed in acacia honey samples from a beekeeper from a town (Table 5). Honey in the 6.25% concentration did not show any antimicrobial activity against *E. faecalis*.

Against *B. subtilis*, antimicrobial activity was found in 8 samples and ranged between 3.00 and 11.67 mm. The best antimicrobial effect was found in multifloral honey samples from a beekeeper from a town (Table 6). In the 6.25% concentration, only one sample showed an antagonistic effect against this microbe which was sample no. 4, multifloral honey from a beekeeper from a town.

Values of the inhibition zones for the 50% honey samples in the tests against yeasts are shown in Table 7, Table 8, Table 9 and Table 10. The antimicrobial activity against *C. albicans* ranged from 0.00 to 7.67 mm. The best antimicrobial activity was found in honey samples no. 1 and 65. No 1. was a multifloral honey from a beekeeper from a town and no. 65 was a sunflower honey from a beekeeper of the countryside. Comparing the activity of honey to antifungal fluconazole, a stronger antimicrobial effect was found in fluconazole (20.33 mm) (Table 7). In 18 samples of the 50% honey, antimicrobial activity against *C. albicans* was not found. 

The antimicrobial activity of the honey samples against *C. glabrata* ranged from 0.00 to 10.00 mm (Table 8). The largest zone of inhibition was found in sample no. 100, phacelia honey from a beekeeper collected in the countryside. A larger antimicrobial effect was found against *C. glabrata* for fluconazole (24.67 mm). Antimicrobial activity against *C. glabrata* was not observed in 19 samples.

The antimicrobial activity of honey samples against *C. krusei* ranged between 0.00 and 9.67 mm. The best antimicrobial activity was found in fruit-tree honey from a beekeeper collected in the countryside. The susceptibility of *C. krusei* to fluconazole was higher (22.33 mm) (Table 9). Ten honey samples did not show any effects against *C. krusei*. 

The inhibition zones of honey samples against *C. tropicalis* ranged from 0.00 to 10.67 mm. The inhibition zone of fluconazole against this yeast was 21.33 mm (Table 10). Susceptibility of *C. tropicalis* to the antifungal compound was stronger than to honey samples. Seven samples of honey were not effective against *C. tropicalis*.

The anticandidal effect of honey in the 50% concentration was found in 82% of honey samples against *C. albicans,* in 81% of honey samples against *C. glabrata*, in 90% of honey samples against *C. krusei*, and in 93% of honey against *C. tropicalis*. Honey samples in 50% concentration were more effective against *Candida* species compared to bacterial species.

The anticandidal effect of the 25% concentration of honey was found for each candida only in the sample no. 100 which was phacelia honey from a beekeeper from a countryside apiary. The sizes of inhibition zones were: 6.33 mm against *C. tropicalis*, 3.33 mm against *C. glabrata*, 1.00 mm against *C. albicans*, and 0.33 mm against *C. krusei*.

### 2.2. Antioxidant Activity of Honey

The level of DPPH inhibition ranged from 8.51% to 70.83% which corresponded to 277.71–2373.85 μg Tx/g TEAC. A stronger activity, higher than 1000 μg Tx/g TEAC, was observed for 13 samples (sample numbers 64, 95, 28, 32, 34, 33, 27, 35, 94, 69, 93, 90, and 81). A very strong activity was determined only in honey sample 64 with 70.83% inhibition which corresponded to 2373.85 μg Tx/g TEAC (Table 11).

With respect to the type of honey, the highest activity was determined in buckwheat honey samples with an average activity of 53.11% of DPPH and 1758.19 μg Tx/g TEAC. A very good activity was also found in the group of honeydew honey samples, where all the samples showed high activity in the range from 29.84 to 41.94% and TEAC ranging from 962.49 to 1354.72 μg Tx/g. One sample of manuka honey showed an activity value of 34.02% which corresponded to 1097.52 μg Tx/g TEAC. The lowest activity was determined in honey samples from acacia where the activity reached levels ranging from 8.51 to 16.96% and 277.71 to 550.98 μg Tx/g TEAC (Table 12).

## 3. Discussion

Antibiotic resistance is a pressing concern for modern healthcare. According to resistance surveillance studies, resistance to frequently used antibiotics is rising. Honey has been valued for its medicinal properties since ancient times [19]. The present study was performed in order to evaluate the antimicrobial potential of honey produced in Slovakia by beekeepers and honey purchased commercially in Slovak markets against six pathogenic bacterial and four yeast species. The strongest antibacterial activity against Gram-negative bacteria of the 50% concentration honey samples was found towards *P. aeruginosa* (17.67 mm). Among the tested Gram-positive bacteria, honey in the 50% concentration was found the most active against *B. subtilis* (13.00 mm). In the case of yeast and the same honey concentration, *C. tropicalis* was the most susceptible (10.67 mm). Overall, the honey samples showed small differences in the results. In the study of Wadi [20], honey was evaluated against 8 clinical isolates including *Staphylococcus aureus*, *Escherichia coli*, *Klebsiella pneumoniae*, *Pseudomonas aeruginosa*, *Proteus vulgaris*, *Salmonella typhi*, *Shigella sonnei*, and methicillin-resistant *Staphylococcus aureus* (MRSA). Raw natural and commercial honey from the study exhibited antibacterial properties against tested Gram-positive and Gram-negative bacteria. 

Our results revealed that both honey from beekeepers and commercial honey showed identical activity towards most of the tested microorganisms and the same results were revealed in the study of Wadi [20]. Our data imply that the connection between antibacterial activity, floral source, and environmental parameters varies by geographical area. Similar results were found in the study by Wahdan [21]. Antibacterial activity of different floral types of honey was examined against a wide range of Gram-positive and Gram-negative bacterial standard organisms and significant antimicrobial activity was proved [22].

In a study that compared undiluted honey and honey diluted at concentrations of 75, 50, 30, and 10%, it was found effective against *S. aureus* and *S. epidermidis* [23]. In our study, where the antimicrobial activity of 100 honey samples diluted to 6.25, 12.5, 25, and 50% concentrations was compared to the effect of three antibiotics (streptomycin, chloramphenicol, and fluconazole), the results were similar. Honey samples with the 25% concentration showed very good antimicrobial potential against Gram-negative bacteria (*P. aeruginosa*). Results of tests performed with the well diffusion method showed the highest antimicrobial activity at 10.67 mm. The best antimicrobial activity of honey at a concentration of 25% against Gram-negative bacteria was found against *B. subtilis* with an inhibition zone of 14.33 mm. Comparative research of antibacterial activity of honey in various concentrations determined better activity compared to commonly used antibiotics against nine pathogens from urine samples [24]. An in vitro study on local Nigerian types of honey showed inhibition activity of tested undiluted and diluted samples against enteropathogenic isolates [25,26]. Three honey samples were obtained from Baghdad and were examined for antibacterial activity. Three concentrations of 100, 70, and 50% were used against different organisms. The highest antibacterial activity of honey was reported in a 100% concentration [27]. The activity of Danish honey was mostly considered due to the hydrogen peroxide content [28]. The difference in antibacterial activity of honey greatly depends on its floral origin [29]. Honey rich in multiflora improves antimicrobial properties against several clinically significant microorganisms. Moreover, it increases its nutritional potential [30]. 

The next used concentrations of honey were 12.5 and 6.25%. Among the tested Gram-negative bacteria, an inhibitory effect of 12.5% honey was found towards *P. aeruginosa* in 12 samples. The honey at a concentration of 6.25% inhibited the growth *P. aeruginosa* only in two samples. Honey at a lower concentration was more effective against Gram-positive bacteria. Similar to the results at higher concentrations, *B. subtilis* was inhibited to the greatest extent. Only a few samples of honey at 6.25% concentration showed an antibacterial effect. Compared to antibiotic gentamicin, honey was found more effective as an antibacterial agent against *Pseudomonas* and *Staphylococcus* strains [31]. A synergistic action of honey and antibiotics was demonstrated, and it was suggested that honey can be given orally with antibiotics [32]. Our study demonstrated very good activity in all concentrations against *P. aeruginosa* and *B. subtilis*. The effect of antibiotics on different microorganisms was higher than that of the honey samples.

The best antimicrobial effect was found for multifloral, linden, chestnut, and honeydew honey samples from beekeepers. Furthermore, some multifloral commercial honey samples showed very good activity. As it was mentioned previously, geographical and botanical sources had pronounced effects on the antibacterial properties of honey. The antibacterial activity of honey is influenced by a variety of factors besides the floral source. Honey could be used as an alternative treatment for chronic wounds and burns as it can inhibit different types of microorganisms which do not respond to conventional antibiotics without side effects [20]. In research by Goślinski et al. [33], honeydew honey showed a total content of polyphenols comparable to manuka honey, which was also much higher than that detected in multifloral and linden honey. In a different study, manuka honey, alongside honeydew honey, showed a stronger antimicrobial effect against Gram-positive than against Gram-negative bacteria. Similar results were found in our study. High antibiotic activity of manuka honey was found against the strains of *S. aureus* and *E. faecalis* in our results. Other researchers also reported that Gram-positive bacteria are more sensitive than Gram-negative microorganisms to the bactericidal activity of honey [4,34].

The antimicrobial property of honey was found to increase with an increasing concentration of honey [35]. This concentration dependency was confirmed in our study. Similar dose-dependent antibacterial activity of honey was also observed by Deng et al. [36] and Ghramh et al. [37] in studies on the antibacterial potential of honey obtained from different nectars. Based on different studies, it is possible to conclude from that variation in antibacterial potential of honey could depend on its botanical and geographical origin, storage conditions, and metabolism of honeybees [37,38,39,40,41].

Honey is rich in polyphenols, vitamins, and enzymes, and shows good antioxidant activity [42]. In our study, the highest antioxidant capacity was found in buckwheat honey with 70.83% inhibition which was equivalent to 2373.85 μg Tx/g honey. A strong activity of buckwheat honey was also determined in [43] where the authors compared 20 samples of buckwheat honey. In [44,45], the authors analyzed honey from different floral sources and found that buckwheat honey shows the best antioxidant properties. It is also known that buckwheat pollen and nectar are rich in antioxidants which is reflected in the antioxidant capacity of this honey [46]. The antioxidant activity of buckwheat honey was also studied in vivo where it was found to increase the antioxidant capacity of human serum [47]. 

Good antioxidant activities were also determined in honeydew types of honey where the highest activity reached 41.94% inhibition equivalent to 1354.72 μg Tx/g honey. Bobis et al. [48] found that the DPPH radical scavenging activity ranged from 47.84 to 62.99%. In another study [49], it was found that the antioxidant activity of honeydew honey was in the range from 40.67 to 64.83% DPPH inhibition which was the highest in comparison to acacia, lime, and sunflower types of honey. The authors also determined that the antioxidant activity positively correlated with the contents of total phenols and total flavonoids. The antioxidant capacity was tested in Spanish honey where the determined radical scavenging activity of honeydew honey was 66.8% on average; for comparison, 28.7% inhibition was reported for nectar honey [50]. The relationship between chemical composition and antioxidant activity was supported by other studies [51,52]. Moreover, in [53] it was stated that vitamins did not contribute to antioxidant capacity, while phenolic acids and flavonoids have an impact on the activity.

On the other hand, acacia samples of honey showed the lowest antioxidant activity in the range 8.51–16.96% and 277.71–550.98 TEAC. In [54], antioxidant activity of only 2.35–11.97% DPPH inhibition was reported. The authors also evaluated the activity of chestnut honey which was found to be in the range 0.95–3.54% inhibition. In our study, the chestnut honey samples showed an activity of 18.06–36.31% of DPPH radical inhibition. In the mentioned report, multifloral honey showed the strongest activity which ranged from 30.43 to 30.94% inhibition. In our study, the multifloral honey did not show better activity compared to the other tested honey samples with the highest activity of 25.22% inhibition.

## 4. Materials and Methods

### 4.1. Bee Honey Samples

One hundred various bee honey samples of different floral origins were obtained in 2020 from different apiaries as well from the local market where they were commercially sold under different brands. The beekeepers determined the floral source of the honey based on the availability of flora for nectar foraging, the location of the apiary, and the organoleptic qualities of the honey. Honey samples were stored in sterile glass jars at room temperature. Samples were labeled according to the source, location, and floral origin as shown in Table 13.

### 4.2. Microorganisms

Gram-negative bacteria (*Pseudomonas aeruginosa* CCM 3955, *Yersinia enterocolitica* CCM 7204, *Salmonella enterica* subsp. *enterica* 4420), Gram-positive bacteria (*Bacillus subtilis* CCM 1999, *Staphylococcus aureus* subsp. *aureus* CCM 2461, *Enterococcus faecalis* CCM 4224), and yeasts (*Candida albicans* CCM 8261, *Candida glabrata* CCM 8270, *Candida krusei* CCM 8271, *Candida tropicalis* CCM 8223) were obtained from the Czech Collection of Microorganisms (Brno, Czech Republic). 

### 4.3. Determination of Antimicrobial Activity

The antimicrobial activity of each honey sample was determined using the well diffusion method. The inoculum was cultured for 24 h on Muller Hinton Broth (MHB, Oxoid, Basingstoke, UK) at 37 °C for bacteria and on Sabouraud Dextrose Broth (SDB, Oxoid, Basingstoke, UK) at 25 °C for yeast. In total, 100 µL of inoculum in a concentration of 0.5 McFarland standard (1.5 × 10^8^ CFU/mL) was applied to a Petri dish (PD) with 20 mL of Mueller Hinton agar (MHA, Oxoid, Basingstoke, UK) for bacteria or Sabouraud Dextrose Agar (SDA, Oxoid, Basingstoke, UK) for yeasts. The following concentrations of the honey solutions were diluted with MHB resp. SDB: 6.25, 12.5, 25, and 50%. Subsequently, wells of 10 mm diameter were made with a sterile borer into agar plates containing the bacterial and yeast inoculum. In total, 20 μL of analyzed honey was added to the wells. The samples were incubated for 24 h at 37 °C for bacteria and 25 °C for yeast. Antibiotics (chloramphenicol, streptomycin, Oxoid, Basingstoke, UK) were used as a positive control for Gram-negative and Gram-positive bacteria. An antifungal (fluconazole, Oxoid, Basingstoke, UK) was used as a positive control for yeast. Disks impregnated with MHB served as a negative control. Inhibition zones were measured from the edge of the well to border of the bacterial growth at three sides. An inhibition zone above 10 mm was determined to be very strong antimicrobial activity, an inhibition zone above 5 mm was determined to be mild activity, and an inhibition zone above 1 mm was determined to be weak activity. Antimicrobial activity was measured three times.

### 4.4. Antioxidant Activity of Honey Samples

The antioxidant activity of honey samples was determined using the DPPH radical method. The DPPH (Sigma Aldrich, Schnelldorf, Germany) solution was prepared in methanol to a stock concentration 0.025 g/L and was adjusted using methanol to an absorbance of 0.8 at 515 nm (Glomax spectrophotometer, Promega Inc., Madison, WI, USA).

The 0.2 g of honey was mixed with 1 mL of distilled water. After that, 20 µL of suspension was added to 180 µL of DPPH solution in a 96-well plate. The samples were incubated on a shaker at 500 rpm for 30 min in the dark. The absorbance was measured at 515 nm. The % inhibition was calculated according to the formula:

$$\%\;of\;inhibition=\frac{A_{control}-A{\;}_{sample}}{A_{control}}\times 100$$

The control sample contained 20 µL of distilled water with 180 µL DPPH. 

The total antioxidant capacity (TEAC) of honey samples was also calculated with the standard reference Trolox (Sigma Aldrich, Schnelldorf, Germany) prepared in methanol to 5 concentrations in the range of 20–100 µg/mL. TEAC was evaluated from the calibration curve as µg of Trolox to 1 g of honey sample. 

## 5. Conclusions

Antimicrobial activity, especially with Gram-negative bacteria, Gram-positive bacteria, and yeasts, suggests that the honey under analysis may have a relevant role as natural antibacterial products that weaken the effects of bacterial infections and contribute to the improvement of food. In our study, four different concentrations of honey were studied. Our results showed that honey samples at 50% concentration had the strongest effect on the growth of yeast from the genus *Candida*. A lower concentration of honey (25%) produced in Slovakia had antibacterial activity against all Gram-negative and Gram-positive bacteria tested. Concentrations lower than 25% had an influence especially on *P. aeruginosa*, *S. aureus*, *E. faecalis*, and *B. subtilis*. The antioxidant activity was the highest in buckwheat honey. The majority of honey samples with good antioxidant properties were of the honeydew type.

## Figures and Tables

**Table 1:** Antimicrobial activity of samples against *S. enterica* in mm.

Sample No.	50%	25%	12.5%	6.25%
1	6.33 ± 1.15	-	-	-
2	6.67 ± 1.53	5.67 ± 0.58	-	-
3	2.67 ± 0.58	-	-	-
4	3.33 ± 0.58	-	-	-
5	3.67 ± 0.58	-	-	-
6	4.33 ± 0.58	-	-	-
7	3.67 ± 0.58	-	-	-
8	5.00 ± 1.00	-	-	-
9	4.33 ± 0.58	-	-	-
10	3.33 ± 0.58	-	-	-
11	3.67 ± 0.58	-	-	-
12	2.67 ± 0.58	-	-	-
13	5.67 ± 0.58	-	-	-
14	4.33 ± 0.58	-	-	-
15	3.33 ± 0.58	-	-	-
16	2.67 ± 0.58	-	-	-
17	5.67 ± 0.58	-	-	-
18	6.67 ± 0.58	-	-	-
21	5.33 ± 1.15	-	-	-
22	4.67 ± 0.58	-	-	-
29	10.33 ± 0.58	-	-	-
30	9.67 ± 0.58	6.00 ± 1.00	-	-
31	10.67 ± 0.58	6.67 ± 0.58	-	-
32	8.67 ± 0.58	7.00 ± 1.00	-	-
33	5.33 ± 0.58	5.67 ± 0.58	-	-
34	5.33 ± 0.58	-	-	-
35	4.33 ± 0.58	-	-	-
36	8.00 ± 1.00	-	-	-
37	4.67 ± 0.58	-	-	-
38	6.33 ± 0.58	-	-	-
39	4.33 ± 0.58	-	-	-
40	5.67 ± 0.58	-	-	-
41	4.33 ± 0.58	-	-	-
42	3.67 ± 0.58	-	-	-
43	4.67 ± 0.58	-	-	-
44	2.67 ± 0.58	-	-	-
45	4.33 ± 0.58	-	-	-
46	3.67 ± 0.58	-	-	-
47	4.33 ± 0.58	-	-	-
48	3.67 ± 0.58	-	-	-
49	3.67 ± 0.58	-	-	-
50	7.67 ± 0.58	-	-	-
51	3.33 ± 0.58	-	-	-
52	2.33 ± 0.58	-	-	-
53	4.67 ± 0.58	-	-	-
54	4.33 ± 0.58	-	-	-
55	5.33 ± 0.58	-	-	-
56	5.67 ± 0.58	-	-	-
57	4.67 ± 0.58	-	-	-
58	5.33 ± 0.58	-	-	-
59	5.67 ± 0.58	-	-	-
60	6.67 ± 0.58	-	-	-
61	7.00 ± 1.00	-	-	-
62	6.67 ± 0.58	-	-	-
63	6.67 ± 0.58	-	-	-
64	7.67 ± 0.58	-	-	-
65	2.33 ± 0.58	-	-	-
66	3.67 ± 0.58	-	-	-
67	3.33 ± 0.58	-	-	-
68	4.33 ± 0.58	-	-	-
69	4.33 ± 0.58	-	-	-
70	7.33 ± 0.58	-	-	-
71	7.67 ± 0.58	-	-	-
72	5.67 ± 0.58	-	-	-
73	3.67 ± 0.58	-	-	-
74	6.00 ± 1.00	-	-	-
75	2.67 ± 0.58	-	-	-
76	2.67 ± 0.58	-	-	-
77	5.67 ± 0.58	-	-	-
78	5.33 ± 0.58	-	-	-
79	4.67 ± 0.58	-	-	-
80	5.33 ± 0.58	-	-	-
81	4.67 ± 0.58	-	-	-
82	3.67 ± 0.58	-	-	-
83	4.33 ± 0.58	-	-	-
84	3.33 ± 0.58	-	-	-
85	4.33 ± 0.58	-	-	-
86	5.00 ± 1.00	-	-	-
87	8.67 ± 0.58	4.67 ± 0.58	-	-
88	10.67 ± 0.58	4.33 ± 0.58	-	-
89	3.33 ± 0.58	-	-	-
90	3.67 ± 0.58	-	-	-
91	3.33 ± 0.58	-	-	-
92	2.67 ± 0.58	-	-	-
93	3.67 ± 0.58	-	-	-
94	4.67 ± 0.58	-	-	-
95	4.67 ± 0.58	-	-	-
96	3.33 ± 0.58	-	-	-
97	6.67 ± 0.58	-	-	-
98	5.33 ± 0.58	-	-	-
99	3.67 ± 0.58	-	-	-
100	3.33 ± 0.58	1.67 ± 0.58	-	-
ATB	20.67 ± 1.15			

**Table 2:** Antimicrobial activity of samples against *Y. enterocolitica* in mm.

Sample No.	50%	25%	12.5%	6.25%
3	2.67 ± 0.58	-	-	-
13	2.33 ± 0.58	-	-	-
14	2.33 ± 0.58	-	-	-
15	2.67 ± 0.58	-	-	-
16	2.00 ± 1.00	-	-	-
17	5.33 ± 0.58	-	-	-
18	5.00 ± 1.00	-	-	-
19	5.00 ± 1.00	-	-	-
20	9.00 ± 1.00	6.67 ± 0.58	-	-
25	5.33 ± 1.53	-	-	-
26	4.67 ± 0.58	-	-	-
27	3.67 ± 0.58	-	-	-
28	3.67 ± 0.58	-	-	-
29	10.33 ± 0.58	8.67 ± 0.58	-	-
30	9.67 ± 0.58	7.67 ± 0.58	-	-
31	10.67 ± 0.58	7.33 ± 0.58	-	-
32	10.67 ± 1.15	7.67 ± 0.58	-	-
33	4.33 ± 0.58	-	-	-
38	9.33 ± 1.15	8.33 ± 1.15	-	-
44	2.33 ± 0.58	-	-	-
45	2.33 ± 0.58	-	-	-
46	2.67 ± 0.58	-	-	-
47	2.33 ± 0.58	-	-	-
48	2.33 ± 0.58	-	-	-
53	4.33 ± 0.58	-	-	-
56	3.67 ± 0.58	-	-	-
57	4.33 ± 0.58	-	-	-
58	4.67 ± 0.58	-	-	-
59	2.67 ± 0.58	-	-	-
60	3.67 ± 0.58	-	-	-
61	10.33 ± 0.58	8.67 ± 0.58	-	-
62	10.67 ± 0.58	8.33 ± 0.58	-	-
63	8.67 ± 1.15	-	-	-
64	7.33 ± 0.58	-	-	-
65	5.67 ± 0.58	-	-	-
66	10.33 ± 0.58	7.67 ± 0.58	-	-
67	11.33 ± 0.58	6.00 ± 1.00	-	-
68	9.33 ± 0.58	5.67 ± 1.15	-	-
77	4.33 ± 0.58	-	-	-
78	5.33 ± 0.58	-	-	-
79	2.33 ± 0.58	-	-	-
80	2.67 ± 0.58	-	-	-
81	4.67 ± 0.58	-	-	-
82	4.67 ± 1.15	-	-	-
83	4.67 ± 0.58	-	-	-
84	5.33 ± 1.15	-	-	-
100	6.33 ± 1.15	6.67 ± 1.53	-	-
ATB	14.67 ± 0.58			

**Table 3:** Antimicrobial activity of samples against *P. aeruginosa* in mm.

Sample No.	50%	25%	12.5%	6.25%
5	7.00 ± 1.00	-	-	-
6	8.67 ± 0.58	-	-	-
7	5.00 ± 0.00	-	-	-
8	6.33 ± 0.58	-	-	-
9	13.33 ± 1.53	9.67 ± 0.58	7.67 ± 0.58	-
10	13.00 ± 1.00	10.33 ± 0.58	8.33 ± 1.15	-
11	13.33 ± 1.53	9.33 ± 0.58	6.67 ± 1.15	-
12	10.67 ± 0.58	8.67 ± 0.58	5.00 ± 1.00	-
32	5.33 ± 0.58	-	-	-
41	5.33 ± 0.58	-	-	-
42	7.67 ± 0.58	-	-	-
43	6.67 ± 0.58	-	-	-
44	5.67 ± 0.58	-	-	-
45	10.33 ± 0.58	-	-	-
46	8.67 ± 0.58	10.33 ± 0.58	6.33 ± 1.15	-
50	5.67 ± 0.58	10.33 ± 0.58	5.67 ± 0.58	-
61	1.33 ± 0.58	-	-	-
62	1.67 ± 0.58	-	-	-
63	11.67 ± 0.58	10.33 ± 0.58	6.00 ± 1.00	-
64	10.33 ± 0.58	10.33 ± 0.58	5.67 ± 0.58	-
65	9.67 ± 0.58	10.00 ± 1.00	6.00 ± 1.00	-
66	6.67 ± 0.58	-	-	-
69	17.67 ± 2.52	10.33 ± 0.58	4.33 ± 0.58	-
70	12.33 ± 0.58	10.67 ± 0.58	5.33 ± 0.58	-
71	5.67 ± 0.58	-	-	-
72	10.33 ± 0.58	-	-	-
73	4.67 ± 0.58	-	-	-
89	5.67 ± 0.58	-	-	-
97	2.67 ± 0.58	-	-	-
98	3.00 ± 1.00	-	-	-
99	2.67 ± 0.58	-	-	-
100	4.67 ± 0.58	6.67 ± 1.53	6.67 ± 1.53	-
ATB	20.33 ± 0.58			

**Table 4:** Antimicrobial activity of samples against *S. aureus* in mm.

Sample No.	50%	25%	12.5%	6.25%
1	8.67 ± 0.58	4.67 ± 0.58		
2	10.33 ± 0.58	5.00 ± 1.00		
3	12.33 ± 0.58	8.67 ± 0.58	3.33 ± 0.58	
4	10.33 ± 0.58	13.00 ± 1.00	10.33 ± 0.58	5.33 ± 0.58
18	8.33 ± 0.58	8.67 ± 0.58	-	-
19	9.00 ± 1.00	9.33 ± 1.15	7.00 ± 1.00	-
53	11.00 ± 1.00	11.00 ± 1.00	9.33 ± 0.58	3.33 ± 0.58
54	11.00 ± 1.00	11.00 ± 1.00	7.33 ± 0.58	-
55	11.67 ± 0.58	10.33 ± 0.58	7.00 ± 1.00	-
56	10.67 ± 0.58	10.67 ± 0.58	6.00 ± 1.00	-
69	11.00 ± 1.00	11.33 ± 0.58	6.33 ± 1.15	-
71	2.67 ± 0.58	-	-	-
72	3.33 ± 0.58	-	-	-
82	3.33 ± 0.58	-	-	-
83	2.33 ± 0.58	-	-	-
84	4.33 ± 0.58	-	-	-
90	8.33 ± 0.58	10.33 ± 0.58	5.33 ± 0.58	-
91	7.67 ± 0.58	-	-	-
100	4.33 ± 0.58	2.33 ± 0.58	-	-
ATB	22.33 ± 0.58			

**Table 5:** Antimicrobial activity of samples against *E. faecalis* in mm.

Sample No.	50%	25%	12.5%	6.25%
5	10.33 ± 0.58	-	-	-
6	10.67 ± 1.15	-	-	-
7	11.33 ± 0.58	-	-	-
8	9.67 ± 0.58	-	-	-
9	5.67 ± 0.58	-	-	-
10	7.67 ± 0.58	-	-	-
11	4.67 ± 0.58	-	-	-
12	6.67 ± 0.58	-	-	-
13	11.67 ± 0.58	10.33 ± 0.58	-	-
14	11.33 ± 0.58	10.00 ± 1.73	-	-
15	11.00 ± 1.00	10.33 ± 0.58	-	-
16	12.33 ± 0.58	11.00 ± 1.00	-	-
17	4.67 ± 0.58	-	-	-
18	5.00 ± 1.00	-	-	-
19	3.67 ± 0.58	-	-	-
20	4.67 ± 0.58	-	-	-
25	4.67 ± 1.15	-	-	-
26	4.67 ± 0.58	-	-	-
27	5.33 ± 0.58	-	-	-
28	6.33 ± 0.58	-	-	-
29	4.00 ± 1.00	-	-	-
30	6.33 ± 0.58	-	-	-
31	4.33 ± 0.58	-	-	-
32	2.67 ± 0.58	-	-	-
33	4.33 ± 0.58	-	-	-
34	5.33 ± 0.58	-	-	-
35	4.67 ± 0.58	-	-	-
36	5.00 ± 1.00	-	-	-
37	8.33 ± 0.58	-	-	-
38	10.33 ± 0.58	-	-	-
39	10.67 ± 0.58	-	-	-
40	11.33 ± 1.15	-	-	-
45	5.67 ± 0.58	-	-	-
46	6.67 ± 0.58	-	-	-
47	5.33 ± 0.58	-	-	-
48	8.33 ± 0.58	-	-	-
49	3.67 ± 0.58	-	-	-
50	4.33 ± 0.58	-	-	-
51	2.67 ± 0.58	-	-	-
52	3.67 ± 0.58	-	-	-
53	7.67 ± 0.58	-	-	-
54	6.67 ± 0.58	-	-	-
55	6.33 ± 0.58	-	-	-
56	5.67 ± 0.58	-	-	-
57	7.67 ± 0.58	-	-	-
58	8.67 ± 0.58	-	-	-
59	7.67 ± 0.58	-	-	-
60	7.33 ± 0.58	-	-	-
65	5.67 ± 0.58	-	-	-
66	4.67 ± 0.58	-	-	-
67	3.67 ± 0.58	-	-	-
68	4.33 ± 0.58	-	-	-
69	4.67 ± 0.58	-	-	-
70	5.33 ± 0.58	-	-	-
71	2.33 ± 0.58	-	-	-
72	2.67 ± 0.58	-	-	-
73	5.67 ± 0.58	-	-	-
74	6.00 ± 1.00	-	-	-
75	5.33 ± 0.58	-	-	-
76	6.33 ± 0.58	-	-	-
77	5.67 ± 0.58	-	-	-
78	4.67 ± 0.58	-	-	-
79	3.33 ± 0.58	-	-	-
80	3.67 ± 0.58	-	-	-
81	4.33 ± 0.58	-	-	-
82	4.33 ± 1.15	-	-	-
83	5.00 ± 1.00	-	-	-
84	4.67 ± 0.58	-	-	-
85	10.67 ± 0.58	-	-	-
86	9.33 ± 0.58	-	-	-
87	9.67 ± 0.58	-	-	-
88	10.33 ± 1.15	-	-	-
89	5.00 ± 1.00	-	-	-
90	4.67 ± 0.58	-	-	-
91	5.33 ± 0.58	-	-	-
92	4.67 ± 1.15	-	-	-
93	4.33 ± 0.58	-	-	-
94	4.67 ± 0.58	-	-	-
95	3.67 ± 0.58	-	-	-
96	3.33 ± 0.58	-	-	-
97	5.67 ± 0.58	-	-	-
98	6.33 ± 0.58	-	-	-
99	5.33 ± 0.58	-	-	-
100	1.67 ± 0.58	-	-	-
ATB	24.67 ± 0.58			

**Table 6:** Antimicrobial activity of samples against *B. subtilis* in mm.

Sample No.	50%	25%	12.5%	6.25%
1	11.00 ± 1.00	11.00 ± 1.00	6.33 ± 0.58	-
2	8.67 ± 0.58	11.33 ± 1.15	8.33 ± 1.15	-
3	5.33 ± 0.58	-	-	-
4	13.00 ± 1.00	14.33 ± 1.53	11.67 ± 0.58	6.67 ± 0.58
5	2.67 ± 0.58	-	-	-
9	5.33 ± 0.58	-	-	-
10	2.33 ± 0.58	-	-	-
11	5.33 ± 0.58	-	-	-
12	6.00 ± 1.00	-	-	-
15	6.67 ± 1.53	-	-	-
16	6.33 ± 1.15	-	-	-
19	4.67 ± 0.58	-	-	-
21	5.33 ± 0.58	-	-	-
22	2.33 ± 0.58	-	-	-
23	9.67 ± 0.58	-	-	-
24	6.67 ± 0.58	-	-	-
25	4.67 ± 0.58	-	-	-
27	5.33 ± 0.58	-	-	-
28	2.33 ± 0.58	-	-	-
30	6.00 ± 1.73	-	-	-
31	6.33 ± 0.58	-	-	-
34	12.67 ± 1.53	9.67 ± 0.58	6.67 ± 0.58	-
35	9.67 ± 0.58	-	-	-
36	4.67 ± 0.58	-	-	-
37	5.00 ± 1.73	-	-	-
39	10.67 ± 1.53	9.67 ± 0.58	4.33 ± 0.58	-
41	9.67 ± 0.58	8.33 ± 0.58	3.00 ± 1.00	-
42	6.67 ± 0.58	-	-	-
43	4.67 ± 0.58	-	-	-
44	4.67 ± 0.58	-	-	-
45	5.33 ± 0.58	-	-	-
46	8.33 ± 0.58	-	-	-
47	6.67 ± 0.58	-	-	-
48	4.67 ± 0.58	-	-	-
51	4.67 ± 0.58	-	-	-
52	3.33 ± 0.58	-	-	-
53	2.33 ± 0.58	-	-	-
54	2.33 ± 0.58	-	-	-
55	3.00 ± 1.00	-	-	-
56	4.33 ± 0.58	-	-	-
57	4.67 ± 0.58	-	-	-
58	4.67 ± 0.58	-	-	-
59	5.67 ± 0.58	-	-	-
60	5.33 ± 0.58	-	-	-
62	6.67 ± 0.58	-	-	-
63	4.33 ± 0.58	-	-	-
64	2.67 ± 0.58	-	-	-
65	3.67 ± 0.58	-	-	-
66	4.33 ± 0.58	-	-	-
67	5.33 ± 0.58	-	-	-
68	5.67 ± 0.58	-	-	-
69	5.67 ± 0.58	-	-	-
70	3.33 ± 0.58	-	-	-
71	4.33 ± 0.58	-	-	-
72	3.33 ± 0.58	-	-	-
73	4.67 ± 0.58	-	-	-
74	4.67 ± 0.58	-	-	-
75	2.33 ± 0.58	-	-	-
76	3.67 ± 0.58	-	-	-
81	2.33 ± 0.58	-	-	-
82	4.67 ± 0.58	-	-	-
83	3.33 ± 0.58	-	-	-
84	2.33 ± 0.58	-	-	-
86	7.67 ± 2.31	-	-	-
87	5.00 ± 1.73	-	-	-
89	4.67 ± 0.58	-	-	-
90	2.67 ± 0.58	-	-	-
91	3.67 ± 0.58	-	-	-
92	2.33 ± 0.58	-	-	-
93	1.67 ± 0.58	-	-	-
94	3.67 ± 0.58	-	-	-
95	4.67 ± 0.58	-	-	-
96	3.33 ± 0.58	10.33 ± 0.58	5.67 ± 0.58	-
97	8.67 ± 0.58	10.33 ± 0.58	5.67 ± 1.15	-
98	4.67 ± 0.58	-	-	-
99	5.67 ± 0.58	-	-	-
100	9.33 ± 0.58	4.33 ± 0.58	-	-
ATB	22.33 ± 0.58			

**Table 7:** Antimicrobial activity of samples against *C. albicans* in mm.

Sample No.	50%	25%	12.5%	6.25%
1	7.67 ± 0.58	-	-	-
2	4.33 ± 0.58	-	-	-
3	3.33 ± 0.58	-	-	-
5	7.33 ± 0.58	-	-	-
6	3.33 ± 0.58	-	-	-
8	2.33 ± 0.58	-	-	-
9	3.33 ± 0.58	-	-	-
10	2.33 ± 0.58	-	-	-
11	4.33 ± 0.58	-	-	-
12	4.67 ± 0.58	-	-	-
13	4.67 ± 0.58	-	-	-
14	2.33 ± 0.58	-	-	-
17	4.67 ± 0.58	-	-	-
21	4.33 ± 0.58	-	-	-
22	2.33 ± 0.58	-	-	-
28	4.67 ± 0.58	-	-	-
29	5.33 ± 0.58	-	-	-
30	2.33 ± 0.58	-	-	-
31	4.33 ± 0.58	-	-	-
32	3.67 ± 0.58	-	-	-
33	3.33 ± 0.58	-	-	-
34	2.33 ± 0.58	-	-	-
35	3.67 ± 0.58	-	-	-
36	4.33 ± 0.58	-	-	-
37	2.33 ± 0.58	-	-	-
41	3.67 ± 0.58	-	-	-
42	2.33 ± 0.58	-	-	-
43	3.67 ± 0.58	-	-	-
44	4.67 ± 0.58	-	-	-
45	4.67 ± 0.58	-	-	-
46	2.33 ± 0.58	-	-	-
47	3.67 ± 0.58	-	-	-
48	6.67 ± 0.58	-	-	-
49	5.67 ± 0.58	-	-	-
50	4.33 ± 0.58	-	-	-
51	6.67 ± 0.58	-	-	-
52	5.67 ± 0.58	-	-	-
53	3.33 ± 0.58	-	-	-
54	4.67 ± 0.58	-	-	-
55	5.33 ± 0.58	-	-	-
56	4.33 ± 0.58	-	-	-
57	5.67 ± 0.58	-	-	-
58	4.33 ± 0.58	-	-	-
59	4.67 ± 0.58	-	-	-
60	4.33 ± 0.58	-	-	-
61	6.67 ± 0.58	-	-	-
62	4.67 ± 0.58	-	-	-
63	5.33 ± 0.58	-	-	-
64	3.67 ± 0.58	-	-	-
65	7.67 ± 0.58	-	-	-
66	2.33 ± 0.58	-	-	-
69	6.67 ± 0.58	-	-	-
70	2.67 ± 0.58	-	-	-
71	5.67 ± 0.58	-	-	-
72	6.33 ± 0.58	-	-	-
73	3.67 ± 0.58	-	-	-
74	2.33 ± 0.58	-	-	-
75	2.33 ± 0.58	-	-	-
76	4.33 ± 0.58	-	-	-
77	4.67 ± 0.58	-	-	-
78	4.67 ± 0.58	-	-	-
79	2.33 ± 0.58	-	-	-
80	5.33 ± 0.58	-	-	-
81	4.67 ± 1.15	-	-	-
82	4.67 ± 0.58	-	-	-
83	3.00 ± 1.00	-	-	-
84	4.33 ± 0.58	-	-	-
85	5.67 ± 0.58	-	-	-
86	3.67 ± 0.58	-	-	-
87	5.67 ± 0.58	-	-	-
88	4.33 ± 0.58	-	-	-
89	5.67 ± 0.58	-	-	-
90	4.33 ± 0.58	-	-	-
91	3.33 ± 0.58	-	-	-
92	5.00 ± 1.00	-	-	-
93	4.67 ± 0.58	-	-	-
95	4.67 ± 0.58	-	-	-
96	3.67 ± 0.58	-	-	-
97	4.33 ± 0.58	-	-	-
98	2.33 ± 0.58	-	-	-
99	5.33 ± 0.58	-	-	-
100	4.67 ± 1.15	1.00 ± 1.00	-	-
ATB	20.33 ± 0.58			

**Table 8:** Antimicrobial activity of samples against *C. glabrata* in mm.

Sample No.	50%	25%	12.5%	6.25%
1	7.33 ± 0.58	-	-	-
2	2.33 ± 0.58	-	-	-
3	3.67 ± 0.58	-	-	-
4	2.33 ± 0.58	-	-	-
5	2.33 ± 0.58	-	-	-
7	3.33 ± 0.58	-	-	-
11	2.33 ± 0.58	-	-	-
13	7.33 ± 0.58	-	-	-
14	2.33 ± 0.58	-	-	-
17	4.67 ± 0.58	-	-	-
18	3.33 ± 0.58	-	-	-
21	4.67 ± 0.58	-	-	-
22	2.67 ± 0.58	-	-	-
23	4.33 ± 0.58	-	-	-
24	3.33 ± 0.58	-	-	-
29	6.67 ± 0.58	-	-	-
30	6.33 ± 1.15	-	-	-
31	8.33 ± 0.58	-	-	-
32	8.67 ± 0.58	-	-	-
34	4.33 ± 0.58	-	-	-
35	5.67 ± 0.58	-	-	-
36	4.67 ± 0.58	-	-	-
37	4.67 ± 0.58	-	-	-
38	2.33 ± 0.58	-	-	-
39	6.67 ± 0.58	-	-	-
40	7.67 ± 0.58	-	-	-
41	4.67 ± 0.58	-	-	-
42	3.33 ± 0.58	-	-	-
43	4.67 ± 0.58	-	-	-
44	5.67 ± 0.58	-	-	-
45	6.67 ± 0.58	-	-	-
46	3.33 ± 0.58	-	-	-
47	5.67 ± 0.58	-	-	-
48	5.67 ± 0.58	-	-	-
49	6.67 ± 0.58	-	-	-
50	4.67 ± 0.58	-	-	-
51	3.33 ± 0.58	-	-	-
52	4.67 ± 0.58	-	-	-
53	5.33 ± 0.58	-	-	-
54	6.33 ± 0.58	-	-	-
55	3.67 ± 0.58	-	-	-
56	2.33 ± 0.58	-	-	-
57	4.67 ± 0.58	-	-	-
58	4.00 ± 1.00	-	-	-
59	4.67 ± 0.58	-	-	-
60	7.67 ± 0.58	-	-	-
62	4.33 ± 0.58	-	-	-
65	4.67 ± 0.58	-	-	-
66	3.33 ± 0.58	-	-	-
67	9.33 ± 0.58	-	-	-
68	6.67 ± 0.58	-	-	-
69	5.33 ± 0.58	-	-	-
70	5.33 ± 1.15	-	-	-
71	8.33 ± 0.58	-	-	-
72	4.67 ± 0.58	-	-	-
73	6.67 ± 0.58	-	-	-
74	3.33 ± 0.58	-	-	-
76	4.67 ± 0.58	-	-	-
77	3.33 ± 0.58	-	-	-
78	2.33 ± 0.58	-	-	-
79	4.33 ± 0.58	-	-	-
80	4.67 ± 0.58	-	-	-
81	3.67 ± 0.58	-	-	-
82	4.67 ± 0.58	-	-	-
83	3.33 ± 0.58	-	-	-
84	5.33 ± 0.58	-	-	-
85	2.67 ± 0.58	-	-	-
86	4.33 ± 0.58	-	-	-
87	3.33 ± 0.58	-	-	-
88	2.67 ± 0.58	-	-	-
89	4.67 ± 0.58	-	-	-
90	2.33 ± 0.58	-	-	-
91	2.33 ± 0.58	-	-	-
92	1.67 ± 0.58	-	-	-
93	6.67 ± 0.58	-	-	-
94	3.33 ± 0.58	-	-	-
95	4.67 ± 0.58	-	-	-
96	2.33 ± 0.58	-	-	-
98	3.67 ± 0.58	-	-	-
99	2.33 ± 0.58	-	-	-
100	10.00 ± 2.00	3.33 ± 0.58	-	-
ATB	24.67 ± 0.58			

**Table 9:** Antimicrobial activity of samples against *C. krusei* in mm.

Sample No.	50%	25%	12.5%	6.25%
1	8.33 ± 0.58	-	-	-
2	2.67 ± 1.15	-	-	-
3	6.67 ± 0.58	-	-	-
4	4.33 ± 0.58	-	-	-
5	4.33 ± 0.58	-	-	-
7	2.33 ± 0.58	-	-	-
8	2.67 ± 0.58	-	-	-
9	2.33 ± 0.58	-	-	-
10	2.33 ± 0.58	-	-	-
11	3.33 ± 0.58	-	-	-
12	2.33 ± 0.58	-	-	-
13	4.67 ± 0.58	-	-	-
14	4.33 ± 0.58	-	-	-
15	6.67 ± 0.58	-	-	-
16	5.33 ± 0.58	-	-	-
17	3.67 ± 0.58	-	-	-
18	6.33 ± 0.58	-	-	-
20	4.67 ± 0.58	-	-	-
21	3.33 ± 0.58	-	-	-
22	4.33 ± 0.58	-	-	-
23	3.67 ± 0.58	-	-	-
24	4.33 ± 0.58	-	-	-
25	5.33 ± 0.58	-	-	-
26	2.33 ± 0.58	-	-	-
27	4.67 ± 0.58	-	-	-
28	6.00 ± 1.00	-	-	-
29	6.67 ± 0.58	-	-	-
30	3.33 ± 0.58	-	-	-
31	5.33 ± 0.58	-	-	-
32	5.67 ± 0.58	-	-	-
34	2.33 ± 0.58	-	-	-
35	6.67 ± 0.58	-	-	-
36	4.67 ± 0.58	-	-	-
37	8.33 ± 0.58	-	-	-
38	5.33 ± 0.58	-	-	-
39	7.33 ± 0.58	-	-	-
40	7.33 ± 0.58	-	-	-
42	5.33 ± 0.58	-	-	-
43	4.33 ± 0.58	-	-	-
44	5.33 ± 0.58	-	-	-
45	3.33 ± 0.58	-	-	-
46	3.67 ± 0.58	-	-	-
47	2.33 ± 0.58	-	-	-
48	2.33 ± 0.58	-	-	-
49	6.67 ± 0.58	-	-	-
50	4.67 ± 0.58	-	-	-
51	5.67 ± 0.58	-	-	-
52	7.67 ± 0.58	-	-	-
53	9.67 ± 0.58	-	-	-
54	4.67 ± 0.58	-	-	-
55	2.33 ± 0.58	-	-	-
56	4.67 ± 0.58	-	-	-
57	4.67 ± 0.58	-	-	-
58	3.67 ± 0.58	-	-	-
59	2.33 ± 0.58	-	-	-
60	5.67 ± 0.58	-	-	-
61	5.67 ± 0.58	-	-	-
62	3.33 ± 0.58	-	-	-
63	5.67 ± 0.58	-	-	-
64	3.33 ± 0.58	-	-	-
65	4.00 ± 1.00	-	-	-
67	5.33 ± 0.58	-	-	-
68	4.33 ± 0.58	-	-	-
69	4.67 ± 0.58	-	-	-
70	4.67 ± 0.58	-	-	-
71	3.67 ± 0.58	-	-	-
72	3.33 ± 0.58	-	-	-
73	2.33 ± 0.58	-	-	-
74	4.33 ± 0.58	-	-	-
75	4.67 ± 0.58	-	-	-
76	5.33 ± 0.58	-	-	-
77	5.33 ± 0.58	-	-	-
78	5.67 ± 0.58	-	-	-
79	4.67 ± 0.58	-	-	-
80	3.33 ± 0.58	-	-	-
81	3.33 ± 0.58	-	-	-
82	4.33 ± 0.58	-	-	-
83	4.67 ± 0.58	-	-	-
84	2.33 ± 0.58	-	-	-
85	3.67 ± 0.58	-	-	-
86	4.33 ± 0.58	-	-	-
87	2.33 ± 0.58	-	-	-
88	4.33 ± 0.58	-	-	-
89	2.33 ± 0.58	-	-	-
90	4.67 ± 0.58	-	-	-
91	3.33 ± 0.58	-	-	-
92	3.67 ± 0.58	-	-	-
97	3.33 ± 0.58	-	-	-
99	5.33 ± 0.58	-	-	-
100	0.67 ± 0.58	0.33 ± 0.58	-	-
ATB	22.33 ± 0.58			

**Table 10:** Antimicrobial activity of samples against *C. tropicalis* in mm.

Sample No.	50%	25%	12.5%	6.25%
1	7.33 ± 0.58	-	-	-
2	3.33 ± 0.58	-	-	-
3	4.67 ± 0.58	-	-	-
4	3.33 ± 0.58	-	-	-
5	4.67 ± 0.58	-	-	-
6	3.67 ± 0.58	-	-	-
7	5.33 ± 0.58	-	-	-
8	3.67 ± 0.58	-	-	-
9	4.67 ± 0.58	-	-	-
10	2.33 ± 0.58	-	-	-
11	3.33 ± 0.58	-	-	-
12	2.33 ± 0.58	-	-	-
13	3.33 ± 0.58	-	-	-
14	2.33 ± 0.58	-	-	-
15	5.33 ± 0.58	-	-	-
16	4.67 ± 0.58	-	-	-
17	3.33 ± 0.58	-	-	-
18	2.33 ± 0.58	-	-	-
19	4.67 ± 0.58	-	-	-
20	3.67 ± 0.58	-	-	-
21	4.00 ± 1.00	-	-	-
22	2.33 ± 0.58	-	-	-
23	4.67 ± 0.58	-	-	-
24	3.33 ± 0.58	-	-	-
25	4.33 ± 0.58	-	-	-
26	3.33 ± 0.58	-	-	-
28	3.67 ± 0.58	-	-	-
29	4.67 ± 0.58	-	-	-
30	4.33 ± 0.58	-	-	-
31	2.67 ± 0.58	-	-	-
32	4.67 ± 0.58	-	-	-
33	4.67 ± 0.58	-	-	-
34	5.67 ± 0.58	-	-	-
35	2.33 ± 0.58	-	-	-
36	3.33 ± 0.58	-	-	-
37	3.33 ± 0.58	-	-	-
38	4.67 ± 0.58	-	-	-
39	4.67 ± 0.58	-	-	-
40	4.67 ± 0.58	-	-	-
41	2.33 ± 0.58	-	-	-
43	5.33 ± 0.58	-	-	-
44	4.67 ± 0.58	-	-	-
45	3.67 ± 0.58	-	-	-
46	3.33 ± 0.58	-	-	-
47	4.33 ± 0.58	-	-	-
48	3.67 ± 0.58	-	-	-
49	4.67 ± 0.58	-	-	-
50	3.67 ± 0.58	-	-	-
51	2.33 ± 0.58	-	-	-
52	5.67 ± 0.58	-	-	-
53	3.67 ± 0.58	-	-	-
54	2.33 ± 0.58	-	-	-
55	7.33 ± 0.58	-	-	-
56	4.33 ± 0.58	-	-	-
57	4.67 ± 0.58	-	-	-
58	5.33 ± 0.58	-	-	-
59	5.67 ± 0.58	-	-	-
60	7.67 ± 0.58	-	-	-
62	4.33 ± 1.15	-	-	-
63	3.33 ± 0.58	-	-	-
64	5.33 ± 0.58	-	-	-
65	4.67 ± 0.58	-	-	-
66	5.67 ± 0.58	-	-	-
67	4.67 ± 0.58	-	-	-
68	7.33 ± 0.58	-	-	-
69	4.67 ± 0.58	-	-	-
70	4.67 ± 0.58	-	-	-
71	4.33 ± 0.58	-	-	-
72	5.67 ± 0.58	-	-	-
73	4.67 ± 0.58	-	-	-
74	2.67 ± 0.58	-	-	-
75	3.67 ± 0.58	-	-	-
79	3.67 ± 0.58	-	-	-
80	3.67 ± 0.58	-	-	-
81	2.33 ± 0.58	-	-	-
82	3.00 ± 1.00	-	-	-
83	2.33 ± 0.58	-	-	-
84	2.33 ± 0.58	-	-	-
85	3.33 ± 0.58	-	-	-
86	2.33 ± 0.58	-	-	-
87	4.33 ± 0.58	-	-	-
88	3.67 ± 0.58	-	-	-
89	4.33 ± 0.58	-	-	-
90	3.33 ± 0.58	-	-	-
91	3.67 ± 0.58	-	-	-
92	2.67 ± 0.58	-	-	-
93	4.67 ± 0.58	-	-	-
95	3.67 ± 0.58	-	-	-
96	4.00 ± 1.00	-	-	-
97	5.67 ± 0.58	-	-	-
98	3.67 ± 0.58	-	-	-
99	3.33 ± 0.58	-	-	-
100	10.67 ± 1.15	6.33 ± 1.15	-	-
ATB	21.33 ± 0.58			

**Table 11:** Antioxidant activity of the honey (samples over 1000 TEAC).

Sample No.	% Inhibition	TEAC (μg Tx/1 g of Honey)	Type
64	70.8 ± 1.2	2373.8 ± 37.7	Buckwheat
95	41.9 ± 0.7	1354.7 ± 23.5	Honeydew
28	39.7 ± 2.3	1280.8 ± 73.5	Honeydew
32	39.6 ± 0.2	1277.6 ± 6.4	Honeydew
34	39.2 ± 0.6	1264.7 ± 19.8	Honeydew
33	38.4 ± 0.1	1239.0 ± 3.2	Honeydew
27	37.4 ± 0.9	1206.8 ± 30.4	Honeydew
35	36.3 ± 1.7	1171.5 ± 53.6	Chestnut
94	36.2 ± 0.6	1168.2 ± 18.4	Honeydew
69	35.4 ± 1.8	1142.5 ± 58.5	Buckwheat
93	34.0 ± 0.3	1097.5 ± 9.6	Manuka
90	31.5 ± 1.5	1017.1 ± 48.9	Mixed
81	31.1 ± 0.8	1004.3 ± 25.3	Mixed

Results of % inhibition and TEAC are presented as mean value ± SD.

**Table 12:** Antioxidant activity according to the honey type.

Type of Honey	n	DPPH Inhibition (%)		TEAC (μg Tx/1 g of Honey)	
		Range	Average	Range	Average
Acacia	11	8.51–16.96	11.71	277.71–550.98	378.54
Phacelia	3	10.45–19.81	16.14	367.73–641.00	532.76
Chestnut	2	18.06–36.31	27.19	586.34–1171.46	878.90
Mustard	1	-	24.03	-	776.02
Linden	9	14.26–24.28	19.28	460.96–785.67	624.56
Manuka	1	-	34.02	-	1097.52
Honeydew	8	29.84–41.94	37.78	962.49–1354.72	1219.29
Fruit tree	5	16.99–20.87	19.27	550.97–676.36	624.92
Creamed rapeseed	1	-	18.00	-	583.13
Buckwheat	2	35.38–70.83	53.11	1142.53–2373.85	1758.19
Rapeseed	6	9.39–22.68	16.95	306.64–734.23	541.33
Sunflower	8	12.07–30.78	21.08	393.45–994.64	682.79
Multifloral	35	10.95–25.22	17.49	354.87–814.60	566.96
Mixed	8	14.04–31.54	23.77	454.53–1017.15	768.39

**Table 13:** Details of the collected honey samples.

Code	Producer	Town/Country	Locality or Market	Type
1	beekeeper	Bratislava/SK	town	multifloral
2	beekeeper	Bratislava/SK	town	multifloral
3	beekeeper	Bratislava/SK	town	multifloral
4	beekeeper	Bratislava/SK	town	multifloral
5	beekeeper	Bratislava/SK	town	multifloral
6	beekeeper	Trnava/SK	town	multifloral
7	beekeeper	Trnava/SK	town	multifloral
8	beekeeper	Trnava/SL	town	multifloral
9	beekeeper	Prešov/SK	town	multifloral
10	beekeeper	Prešov/SK	town	multifloral
11	beekeeper	Košice/SK	town	multifloral
12	beekeeper	B. Bystrica/SK	town	multifloral
13	beekeeper	Žilina/SK	town	multifloral
14	beekeeper	Nitra/SK	town	acacia
15	beekeeper	Nitra/SK	town	linden
16	beekeeper	Zvolen/SK	town	multifloral
17	beekeeper	Zvolen/SK	town	multifloral
18	beekeeper	Zvolen/SK	town	linden
19	beekeeper	Pezinok/SK	town	multifloral
20	beekeeper	Prievidza/SK	town	multifloral
21	beekeeper	Prievidza/SK	town	multifloral
22	beekeeper	Kremnická lesy/SK	forest	linden
23	beekeeper	B. Štiavnica/SK	forest	multifloral
24	beekeeper	Štrbské pleso/SK	forest	multifloral
25	beekeeper	Kraskovo/SK	forest	multifloral
26	beekeeper	Sabinov/SK	forest	multifloral
27	beekeeper	Sabinov/SK	forest	honeydew
28	beekeeper	Nitra/SK	town	honeydew
29	beekeeper	Kremnica/SK	forest	honeydew
30	beekeeper	Poltár/SK	forest	multifloral
31	beekeeper	Detva/SK	forest	multifloral
32	beekeeper	Detva/SK	forest	honeydew
33	beekeeper	Senec/SK	countryside	honeydew
34	beekeeper	Levoča/SK	forest	honeydew
35	beekeeper	Choča/SK	forest	chestnut
36	beekeeper	Oponice/SK	forest	chestnut
37	beekeeper	Senec/SK	countryside	multifloral
38	beekeeper	Senec/SK	countryside	phacelia
39	beekeeper	Choča/SK	countryside	rapeseed
40	beekeeper	Choča/SK	countryside	acacia
41	beekeeper	Hlohovec/SK	countryside	sunflower
42	beekeeper	Oponice/SK	countryside	sunflower
43	beekeeper	Oponice/SK	countryside	acacia
44	beekeeper	Oponice/SK	countryside	rapeseed
45	beekeeper	Šala/SK	countryside	rapeseed
46	beekeeper	Šala/SK	countryside	sunflower
47	beekeeper	Šala/SK	countryside	acacia
48	beekeeper	Levice/SK	countryside	acacia
49	beekeeper	Levice/SK	countryside	sunflower
50	beekeeper	Levice/SK	countryside	phacelia
51	beekeeper	Krupina/SK	countryside	linden
52	beekeeper	Krupina/SK	countryside	acacia
53	beekeeper	Krupina/SK	countryside	fruit trees
54	beekeeper	Kysucké Nové mesto/SK	town	acacia
55	beekeeper	Kysucké Nové mesto/SK	town	fruit trees
56	beekeeper	Kysucké Nové mesto/SK	town	sunflower
57	beekeeper	Záhorie/SK	countryside	linden
58	beekeeper	Záhorie/SK	countryside	rapeseed
59	beekeeper	Záhorie/SK	countryside	sinapis
60	beekeeper	Liptov/SK	forest	multifloral
61	beekeeper	Horná Streda/SK	town	multifloral
62	beekeeper	Senec/SK	countryside	rapeseed
63	beekeeper	Senec/SK	countryside	sunflower
64	beekeeper	Nitra/SK	countryside	buckwheat
65	beekeeper	Kolíňany/SK	countryside	sunflower
66	beekeeper	Kolíňany/SK	countryside	fruit trees
67	beekeeper	Hlohovec/SK	countryside	multifloral
68	beekeeper	Hlohovec/SK	countryside	multifloral
69	beekeeper	Šamorín/SK	countryside	buckwheat
70	beekeeper	Šamorín/SK	countryside	linden
71	beekeeper	Šamorín/SK	countryside	fruit trees
72	beekeeper	Kysucké Nové mesto/SK	countryside	sunflower
73	beekeeper	Kysucké Nové mesto/SK	countryside	acacia
74	beekeeper	Kysucké Nové mesto/SK	countryside	rapeseed
75	beekeeper	Kysucké Nové mesto/SK	countryside	linden
76	commercially	SK	CBA—private label	multifloral
77	commercially	EU—outside EU	LIDL	multifloral
78	commercially	EU—outside EU	COOP	multifloral
79	commercially	SK	COOP	acacia
80	commercially	SK	COOP	multifloral
81	commercially	SK	COOP	multifloral
82	commercially	SK	COOP	multifloral
83	commercially	SK	COOP—private label	multifloral
84	commercially	EU—outside EU	COOP	linden
85	commercially	SK	Billa	acacia
86	commercially	EU—outside EU	Billa—private label	multifloral
87	commercially	SK	Billa	creamed rapeseed
88	commercially	SK	Tesco	linden
89	commercially	EU—outside EU	Tesco	multifloral
90	commercially	EU—outside EU	Tesco	multifloral
91	commercially	EU	Kraj	multifloral
92	commercially	EU—outside EU	Kraj	multifloral
93	commercially	New Zealand	Ceramel	manuka
94	commercially	Turkey	Kaufland	honeydew
95	commercially	EU—outside EU	Kaufland	honeydew
96	commercially	EU—outside EU	Kaufland	fruit trees
97	commercially	SK	Kaufland	acacia
98	commercially	SK	Kaufland	multifloral
99	commercially	EU	Kaufland	multifloral
100	beekeeper	SK	countryside	phacelia

## Data Availability

Not applicable.
